# Increased Scan Speed and Pitch on Ultra-Low-Dose Chest CT: Effect on Nodule Volumetry and Image Quality

**DOI:** 10.3390/medicina60081301

**Published:** 2024-08-12

**Authors:** Heejoo Bae, Ji Won Lee, Yeon Joo Jeong, Min-Hee Hwang, Geewon Lee

**Affiliations:** 1 Department of Radiology and Medical Research Institute, Pusan National University Hospital, Pusan National University School of Medicine, Busan 49241, Republic of Koreamonophobia00@hanmail.net (J.W.L.); hmh8807@naver.com (M.-H.H.); 2 Department of Radiology and Medical Research Institute, Yangsan Pusan National University Hospital, Pusan National University School of Medicine, Busan 50612, Republic of Korea; jeongyj0610@gmail.com

**Keywords:** multidetector computed tomography, pitch, motion artifact, noise, thorax

## Abstract

*Background and Objectives*: This study’s objective was to investigate the influence of increased scan speed and pitch on image quality and nodule volumetry in patients who underwent ultra-low-dose chest computed tomography (CT). *Material and Methods*: One hundred and two patients who had lung nodules were included in this study. Standard-speed, standard-pitch (SSSP) ultra-low-dose CT and high-speed, high-pitch (HSHP) ultra-low-dose CT were obtained for all patients. Image noise was measured as the standard deviation of attenuation. One hundred and sixty-three nodules were identified and classified according to location, volume, and nodule type. Volume measurement of detected pulmonary nodules was compared according to nodule location, volume, and nodule type. Motion artifacts at the right middle lobe, the lingular segment, and both lower lobes near the lung bases were evaluated. Subjective image quality analysis was also performed. *Results*: The HSHP CT scan demonstrated decreased motion artifacts at the left upper lobe lingular segment and left lower lobe compared to the SSSP CT scan (*p* < 0.001). The image noise was higher and the radiation dose was lower in the HSHP scan (*p* < 0.001). According to the nodule type, the absolute relative volume difference was significantly higher in ground glass opacity nodules compared with those of part-solid and solid nodules (*p* < 0.001). *Conclusion*: Our study results suggest that HSHP ultra-low-dose chest CT scans provide decreased motion artifacts and lower radiation doses compared to SSSP ultra-low-dose chest CT. However, lung nodule volumetry should be performed with caution for ground glass opacity nodules.

## 1. Introduction

The widespread availability of multidetector chest CT has led to a significant rise in the detection of pulmonary nodules [1]. Nevertheless, the task of distinguishing early lung cancer from benign nodules is still a hurdle, making chest CT surveillance an essential component of lung nodule management [2,3]. Many clinical trials of lung cancer screening have integrated nodule volumetry into their protocols, relying on nodule size and growth as the primary factors for determining the nature of pulmonary nodules [4,5]. The latest nodule management guidelines of the Fleischner Society now include lung nodule volume definitions, and the British Thoracic Society advocates the use of lung nodule volumetry to define nodule growth [6,7]. Therefore, accurately measuring the volume of an indeterminate pulmonary nodule is crucial for determining its potential growth or stability.

CT scanners continue to advance, with new developments including wider detector coverage, faster gantry rotation, and the use of high-pitch techniques. Theoretically speaking, increased pitch can extend rotation time and decrease scan time, ultimately improving *z*-axis resolution, reducing partial volume effects, and potentially increasing accuracy in volumetry [8]. Two prior investigations have shown that the use of the high pitch was equivalent to the standard pitch in the 3D volumetry of solid lung nodules [9,10]. However, both studies were conducted as phantom experiments that investigated only solid nodules using a 16-section or 128-section scanner [9,10]. In terms of lung parenchyma, Baumueller et al. found that the high-pitch mode provided better image quality compared to a standard spiral mode during breath-holding [11]. According to Schulz et al., the lung parenchyma and vascular structures could potentially be better evaluated using a high-pitch, dual-source mode for patients with poor breath-holding compliance [12]. By increasing from 16 to 128 slices, this study proved a significant impact in reducing the number and severity of motion artifacts. With the introduction of 512-slice multidetector CT scanners, it is now possible to acquire data of the entire chest in less than 1 s through high-pitch scanning [13]. Therefore, the purpose of this study was to investigate the effects of higher scan speed and pitch on image quality and the volumetric measurements of lung nodules in ultra-low-dose chest CT in comparison with the standard parameters.

## 2. Material and Methods

This retrospective study used a prospectively collected database of CT images obtained between December 2017 and September 2018. During the study period, patients who underwent non-enhanced ultra-low-dose chest CT scans at the respiratory center of our institute were included. From this database, patients with nodules who were eligible for volumetric measurement were exclusively selected for our study. Institutional review board (IRB no. 2308-007-129) approval was obtained for this retrospective study with a waiver of informed consent.

### 2.1. Patients

A total of 135 patients (91 males and 44 females) were included in this study. All patients had at least one nodule eligible for automatic nodule segmentation and subsequent volume assessment. During the process of nodule segmentation, patients were excluded if automatic segmentation was judged to be poor due to the following reasons: (1) low contrast between the lung and ground glass opacity (GGO) or (2) attachment to adjacent pleura or vessels. Thus, 33 patients (23 males and 10 females) were excluded, and the final study group included 102 patients (68 males and 34 females).

Chest CT examinations were performed for the following diseases: nodule follow-up (*n* = 38), malignancy (*n* = 31), pulmonary infection (*n* = 24), chronic obstructive pulmonary disease (*n* = 4), bronchiectasis (*n* = 3), and bronchial asthma (*n* = 2). Clinical information was recorded by reviewing electronic charts.

### 2.2. CT Parameters

All patients underwent both standard-speed, standard-pitch (SSSP) and high-speed, high-pitch (HSHP) ultra-low-dose chest CT scans. The SSSP scan was taken first, immediately followed by the HSHP scan while the same position in the CT gantry was maintained with a separate breath hold. Images were obtained from the level of the lung bases to the lung apex. All CT examinations were obtained in the supine position with end-inspiratory breath-holding, and no contrast material was used. The CT images were obtained with 256-slice CT (Revolution CT; GE Healthcare, Waukesha, WI, USA) using the following parameters (SSSP/HSHP): tube voltage, 120 kV/120 kV; tube current, 30–80 mAs; and rotation time, 0.5/0.28; pitch, 0.98/1.53. The reconstruction slice thickness was 2.5 mm. All images were reconstructed using a lung kernel and adaptive statistical iterative reconstruction Veo (ASIR-V) at 50% strength. All reconstructed images were transferred to a dedicated workstation (Advantage Workstation 3.2; GE Healthcare) for volumetry analysis by a radiologist.

The dose-length product was retrieved from the dose report that summarized the individual radiation exposure parameters for each CT scan in the picture archiving and communication system. The effective dose was calculated by applying a method proposed by the European Working Group for Guidelines on Quality Criteria for CT, using the DLP and the conversion coefficient of 0.017 mSv/(mGy·cm) for the chest [14].

### 2.3. Objective Image Quality and Nodule Volumetric Analysis

Unaware of the clinical information, a radiologist with 16 years of experience in thoracic imaging performed the quantitative measurement. First, for assessment of image noise, an axial scan image (2.5 mm thickness) at the level of tracheal bifurcation was selected. The radiologist placed a circular region of interest (ROI) inside the trachea lumen. This area did not include any wall component or secretion. The radiologist obtained the mean attenuation (HU, Hounsfield unit) and image noise (SD, standard deviation) from each ROI [15]. By averaging 102 different noise values (SD) from 102 patients, the mean image noise was quantified.

Next, lung nodules were detected on axial CT scans with a window width of 1500 HU and a level of −700 HU. The nodule location (left upper, left lingular division, left lower, right upper, right middle, and right lower lobe) and nodule type (solid, part-solid, GGO) were recorded. Then, to measure the nodule volume using the workstation, the nodule was manually marked with a mouse click in the nodule’s center and the software program automatically segmented the nodule margin, and CT variables of max 2D diameter, short axis, 3D volume, mean attenuation (HU), and SD were extracted.

### 2.4. Subjective Image Quality Analysis

Two readers, one with 2 years and the other with 16 years of experience in chest CT, participated in the reading process of subjective image quality analysis. The readers were unaware of the protocol or clinical data and were also uninformed of the other radiologist’s scoring. After the independent readings, in cases of controversy, the images were reviewed in consensus by the two radiologists. First, both radiologists assessed all CT scans for motion artifacts. Motion artifacts at the right middle lobe and left upper lobe lingular segment, near the cardiac borders, and both lung bases near the diaphragm were assessed using a four-point scale 1: excellent image quality without visible object distortion or image blurring; 2, good image quality with minor object distortion or blurring; 3, diagnostically partially not acceptable image quality or intermediate blurring; and 4, diagnostically not acceptable image quality with severe object distortion or blurring [16]. Next, the same two readers evaluated nodule detection, nodule contours, streak artifacts, and overall diagnostic acceptability for all CT scans. The grading scale for subjective image quality analysis is provided in Supplementary Table S1.

### 2.5. Statistical Analyses

All continuous data were expressed as means ± SD. The continuous variables were compared using Student’s t-test for normally distributed variables or the Mann–Whitney test for abnormally distributed variables. The categorical variables were compared using the chi-square test or Fisher’s exact test, as appropriate. Cohen’s kappa analysis was used to assess inter-observer agreement in terms of motion artifacts. A κ value of <0.20 was considered to indicate slight agreement; a κ value of 0.21–0.40, fair agreement; a κ value of 0.41–0.60, moderate agreement; a κ value of 0.61–0.80, substantial agreement; and a κ value of ≥0.81, excellent agreement. Volumetric nodule measurements were compared using the Bland–Altman method of assessing agreement [17]. We assumed that the mean of the two measured volumes from the HSHP scan and SSSP scan (VH + VS/2, Vmean) represented the true nodule volume, in which VH represents the 3D volume measurement in the HSHP scan and VS represents the 3D volume measurement in the SSSP scan. Relative volume difference (RVD) was calculated by the following formula: {(VH − VS)/Vmean} × 100, %.

In addition, the values of the RVD (%) were converted to absolute values (removing negative signs), thus defined as the absolute value of the RVD (aRVD) (%), and were compared for nodule location (upper, middle, lower), nodule volume (<500 mm^3^, 500–1000 mm, >1000 mm), and nodule type (GGO, part-solid, solid) using analysis of variance with Tukey’s test. All statistical calculations were performed with SPSS (version 26.0; IBM SPSS Statistics, Armonk, NY, USA) and MedCalc Statistical Software (version 20.027; MedCalc Software Ltd., Ostend, Belgium), and a *p*-value of <0.05 was considered statistically significant.

## 3. Results

### 3.1. Patient and Nodule Characteristics

The demographic characteristics of the 102 patients and the nodule characteristics of the 163 nodules are listed in Table 1.

A total of 163 nodules were identified. Considering nodule location, there were 65 nodules (39.9%) in the upper lobe areas (40 in the right upper lobe and 25 in the left upper lobe apicoposterior and anterior segment), 16 nodules (9.8%) in the middle lobe areas (12 in the right middle lobe and 4 in the left upper lobe lingular division), and 82 nodules (50.3%) in both lower lobes (43 in the right lower lobe and 39 in the left lower lobe). In terms of nodule type, there were 26 GGO nodules (16.0%), 47 part-solid nodules (28.8%), and 90 solid nodules (55.2%). The mean values for maximum 2D diameter, short axis diameter, nodule attenuation, and nodule volume are summarized in Supplementary Table S2.

**Figure 1:**
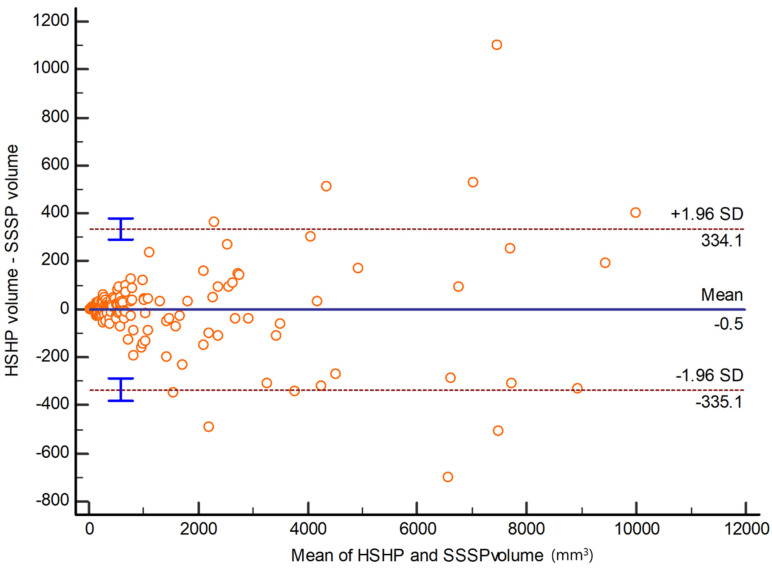
Bland-Altman plots for absolute differences of nodule volumes measured in SSSP and HSHP scans.

**Figure 2:**
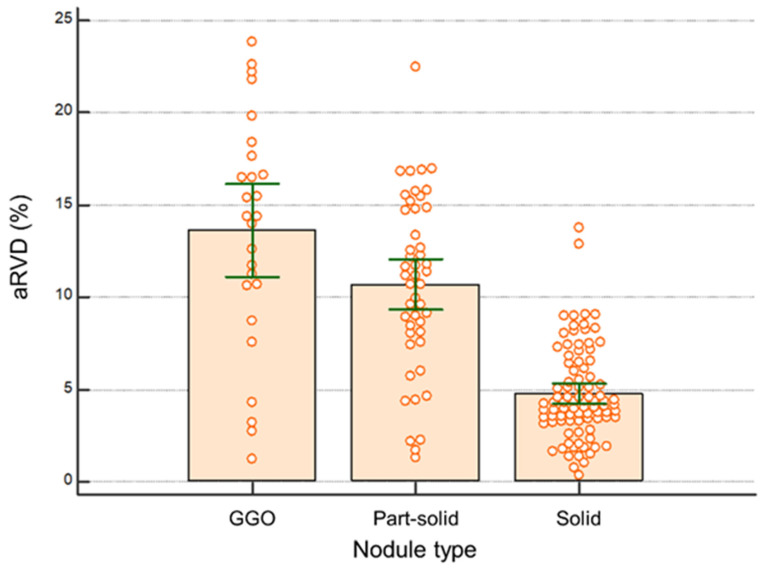
Comparison of aRVD (%) between the SSSP scan and HSHP scan according to nodule type. The error bars represent 95% confidence intervals and the box represents values from the 1st to 3rd quartiles.

The nodule volumes ranged from 26.2 to 10,200 mm^3^ (mean, −158.4; SD, 224.5) on HSHP CT scans and 27.1 to 9800 mm^3^ (mean, −158.2; SD, 226.1) on SSSP CT scans (Figure 1). The mean volume difference between the HSHP and SSSP was 89.3 ± 11.4 mm^3^ (95% CI, 67.6–110.7 mm^3^). The mean aRVD was 7.9 ± 5.4% (range, 0.3–23.8%) for all nodules (Figure 2).

### 3.2. Objective Image Quality between SSSP and HSHP

Table 2 summarizes the image noise and radiation dose between the SSSP and HSHP scans. The HSHP scan showed a significantly decreased radiation dose and increased image noise compared to the SSSP scan.

### 3.3. Volumetry According to Nodule Classification

Table 3 demonstrates a comparison of aRVD (%) between the SSSP and HSHP scans according to nodule location, type, and volume. There was no significant difference in aRVD in terms of nodule location (*p* = 0.486) and nodule volume (*p* = 0.177). According to the nodule type, the aRVD was significantly higher in the GGO nodules compared with those of the part-solid and solid nodules (*p* < 0.001).

### 3.4. Subjective Image Quality Analysis between SSSP and HSHP

**Figure 3:**
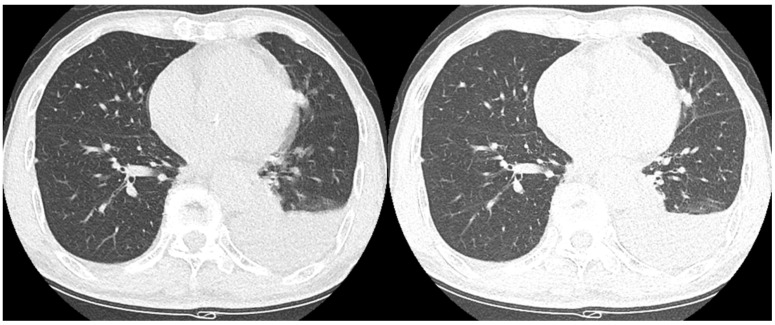
Representative images of decreased motion artifacts in an 81-year-old man. The margin of the solid nodule in the lingular division of the left upper lobe is blurred due to the motion artifacts of the SSSP scan (**left**), whereas the margin is more clearly defined in the HSHP scan (**right**).

Comparisons of the motion artifacts between the SSSP and HSHP scans are shown in Table 4 and Figure 3. The motion artifacts were significantly less in the HSHP scan at the lingular division and left lung base compared to the SSSP scan. The inter-observer agreement for the motion artifacts was moderate to excellent, with kappa values of 0.609 to 0.807.

**Figure 4:**
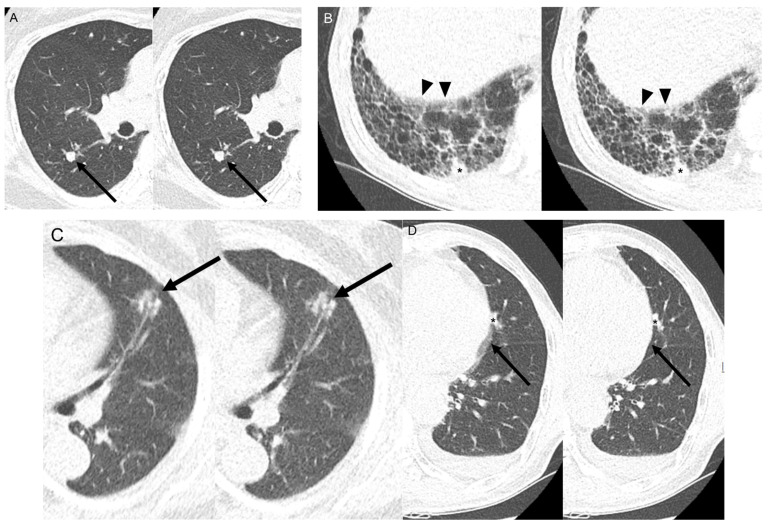
Representative images of motion artifacts and nodule subjective image quality scales between SSSP and HSHP. (**A**) The solid nodule in the right lower lobe (arrow) demonstrates completely confident detection (scale 1) and the completely clear edges (scale 1) of the nodule contours in both the SSSP (**left**) and HSHP images (**right**). The motion artifacts were rated scale 1, of excellent image quality without visible image blurring, for both scans. (**B**) The solid nodule in the right lower lobe (asterisks) shows completely confident detection (scale 1) and the completely clear edges (scale 1) of the nodule contours in both the SSSP (**left**) and HSHP images (**right**). However, in terms of motion artifacts, the SSSP scans show minor blurring of the diaphragm (arrowheads), scoring scale 2, while the HSHP scans show decreased blurring of the diaphragm (arrowheads), scoring scale 1. (**C**) The part-solid nodule in the lingular division shows the average confidence detection (scale 2) and average edge (scale 2) of the nodule contours in both the SSSP (**left**) and HSHP images (**right**). (**D**) The solid nodule (asterisks) in the lingular division shows the poor confidence of detection (scale 3) and less clear edge (scale 3) in the SSSP scan (**left**) and the average confidence of detection (scale 2) and completely clear edge (scale 1) in the HSHP scan (**right**). The motion artifacts were rated scale 3 (intermediate blurring) in the SSSP scan and scale 1 (without image blurring) in the HSHP scan. SSSP = standard-speed, standard-pitch; HSHP = high-speed, high-pitch.

Table 5 shows the results of subjective image quality analysis for nodule detection, contour, streak artifact, and overall image diagnostic acceptability. There were no significant differences in the subjective image quality scores for nodule detection, contour, streak artifact, and overall image diagnostic acceptability. The Kappa values between the two readers for subjective analysis were of substantial to excellent agreement, ranging from 0.636 to 0.917. Representative images of the motion artifacts and subjective image quality analysis of the nodules in the SSSP and HSHP scans are shown in Figure 4.

## 4. Discussion

In our study, the HSHP CT scan demonstrated decreased motion artifacts at the left upper lobe lingular segment and left lung base compared to the SSSP CT scan. The subjective image quality was not different between the two scans. An important advantage of high-pitch CT is reduction in the scan time, which may lead to decreased motion artifacts. Bauer et al. reported that high-pitch dual-source CT pulmonary angiography effectively produced images without respiratory or cardiac motion artifacts even in freely breathing patients [18]. Lell et al. demonstrated that due to a significant reduction in motion artifacts, image quality was superior with high pitch in chest CT scans of children with similar radiation exposure compared to conventional-pitch CT [19]. In a study using submillisievert chest CT for diagnosis of coronavirus disease 2019 infection, the authors applied a relatively high pitch to limit motion artifacts in dyspnea patients [20]. Clinically, our study strengthens the results of previous studies suggesting that high pitch decreases motion artifacts and may potentially help in the scanning of patients with impaired breath-holding.

The radiation dose was lower and the image noise was higher in the HSHP scan in this study. Our results corroborate those of a previous study, which also reported increased noise by using high pitch and similar radiation doses between high-pitch and conventional-pitch CT scans using lung nodule phantoms [10]. Baumueller et al. reported that high pitch was associated with lower radiation doses, but by lowering the dose, the image noise was increased [11]. Generally, decreasing the scan time will lower the radiation dose, but reducing the radiation dose will inevitably increase the image noise, which reduces image quality and spatial resolution [21]. However, the noise difference was not large between the two protocols, and we think the reason is that increasing the pitch selection also resulted in a corresponding increase in the tube current, which likely happened to maintain consistent noise levels in the clinical image [22]. Decreasing the radiation dose is beneficial, as the risks associated with the exposure of patients to ionizing radiation are well-established. This might be particularly helpful for patients who require multiple scans to monitor lung nodules for an extended period of time. Furthermore, there was no difference in subjective image quality with regard to streak artifact and diagnostic acceptability, suggesting that the noise increase was not at a clinically significant level.

Although there was no significant difference in aRVD in terms of nodule location and volume, the aRVD was significantly higher in the GGO nodules compared with those of the part-solid and solid nodules. This may be largely expected because solid nodules have high contrast while GGO nodule margins are more ambiguous, which makes them more able to be influenced by CT techniques. Furthermore, the increased noise in the HSHP scans may have attributed to greater variability in the GGO nodules. The increased noise of low-dose CT images may simulate GGO and interfere with the margins of a subsolid nodule. As a result, the nodule margin is more likely to be blurred and affect nodule segmentation, subsequently leading to inaccurate measurement and nodule misinterpretation [23]. Many studies have reported that the volumetry of GGO nodules is more challenging and the absolute volumetric error values for GGO nodules were higher than those for solid nodules [24,25]. Furthermore, radiation dose and type of iterative reconstruction have also been reported to have a greater effect on GGO nodule measurement accuracy than those of solid nodules [26].

The maximum aRVD observed in this study was 23.8%, falling below the clinically significant level of 25%. The British Thoracic Society and Dutch–Belgian lung-cancer screening trial (Nederlands–Leuvens Longkanker Screenings Onderzoek [NELSON]) utilize a 25% increase in nodule volume as the threshold for determining true nodule growth [27,28]. Our study results fall within the British Thoracic Society and NELSON guidelines, which were established through multiple “coffee break” studies.

There are some limitations to this study. First, we could not accurately discriminate the influence of high pitch from other combined effects such as patient inspiratory effort or cardiovascular pulsation. Second, inherent errors, such as variations in nodule volumetry measurement, may exist. However, we tried to minimize these errors by using the same volumetry software without any manual correction. Third, we only used a single CT scanner with its vendor-specific workstation; hence, our results may have been different if using different software packages that may have yielded variable volumetry results. Fourth, due to the retrospective nature of this study, we were unable to acquire a standard-dose CT scan, which could have been used as a reference. Fifth, we could not compare the estimated nodule volume with the true volume, as there is no ground truth, and even after nodule excision, the degree of lung inflation and pathology techniques may influence nodule size. However, in lung nodule management, the purpose of volumetry is to detect growth rather than absolute nodule size per se.

## 5. Conclusions

In conclusion, our study results suggest that high-pitch and high-speed CT ensures fewer motion artifacts and a lower radiation dose compared with those of standard pitch and standard speed. Despite the increased image noise in the HSHP scans, the volumetry was comparable between the two CT scans. However, the volumetry of the lung nodules varied more in the GGO nodules, suggesting caution when evaluating subsolid nodule volumetry.

## Figures and Tables

**Table 1:** Demographic characteristics of 102 patients and nodule characteristics of 163 evaluated nodules.

Patients (n = 102)	
Age (years) (mean ± SD)	72.3 ± 12.0
Sex (%)	
Male	68 (66.7%)
Female	34 (33.3%)
Nodule characteristics (n = 163)	
Location	
Upper lobes	65 (39.9%)
Middle lobes *	16 (9.8%)
Lower lobes	82 (50.3%)
Type	
Ground glass opacity	26 (16.0%)
Part-solid	47 (28.8%)
Solid	90 (55.2%)
Nodule volume (mm^3^)	
<500	81 (49.7%)
500–1000	29 (17.8%)
>1000	53 (32.5%)

Note—SD = Standard deviation. * Includes the right middle lobe and the left lingular division.

**Table 2:** Comparison of mean image noise and radiation dose between SSSP and HSHP.

Location	SSSP (n = 102 Patients)	HSHP (n = 102 Patients)	*p*-Value
Image noise * (Hounsfield unit)	59.7 ± 5.8 (49.3–70.2)	65.6 ± 5.7 (55.3–75.1)	**<0.001**
Radiation dose (mSv)	0.42 ± 0.30 (0.37–0.48)	0.36 ± 0.33 (0.30–0.42)	**<0.001**

Note—Values are given as means ± standard deviation. Values in parentheses represent 95% confidence intervals. Bold indicates statistical significance at <0.05. SSSP = standard-speed, standard-pitch; HSHP = high-speed, high-pitch. * Image noise was measured by the SD values of ROIs placed in the trachea of each image set.

**Table 3:** Comparison of aRVD (%) between SSSP and HSHP according to nodule location, type, and volume.

Nodule Classification (n = 163)	aRVD	*p*-Value
Nodule location		0.486
Upper lobes	7.3 ± 5.6 (5.9–8.7)	
Middle lobes *	9.0 ± 5.9 (5.8–12.1)	
Lower lobes	8.1 ± 5.1 (7.0–9.2)	
Nodule type		**<0.001**
Ground glass opacity	13.62 ± 6.3 (11.1–16.2)	GGO vs. part-solid **0.009**
Part-solid	10.7 ± 4.7 (9.3–12.1)	Part-solid vs. solid **<0.001**
Solid	4.8 ± 2.5 (4.2–5.3)	GGO vs. solid **<0.001**
Nodule volume (mm^3^)		0.177
<500	8.3 ± 5.0 (7.2–9.4)	
500–1000	8.6 ± 6.1 (6.3–11.0)	
>1000	6.8 ± 5.5 (5.2–8.3)	

Note—Data are expressed as means ± standard deviation. * The middle lobes include the right middle lobe and the lingular division of the left upper lobe. Values in parentheses represent 95% confidence intervals. Bold indicates statistical significance at <0.05. aRVD = absolute value of relative volume difference, GGO = ground glass opacity.

**Table 4:** Comparison of motion artifacts between SSSP and HSHP.

Location/Score	SSSP (n = 102)				HSHP (n = 102)				*p*-Value
	1	2	3	4	1	2	3	4	
Right middle lobe	79	21	2	0	86	15	1	0	0.203
Lingular division	45	54	3	0	66	34	2	0	**0.005**
Right lung base	82	17	3	0	85	15	2	0	0.545
Left lung base	44	54	4	0	64	34	4	0	**0.015**

Note—Data are the number of patients corresponding to each score. The right middle lobe and lingular division were evaluated near the heart border. Bold indicates statistical significance at <0.05. HSHP = high-speed, high-pitch; SSSP = standard-speed, standard-pitch.

**Table 5:** Subjective image quality scores in SSSP and HSHP.

Subjective Analysis		SSSP	HSHP	*p*-Value
Nodule (n = 163 nodules)	Detection	1.07 ± 0.30	1.06 ± 0.27	0.989
	Contour	1.31 ± 0.67	1.22 ± 0.58	0.162
Image	Streak artifact	1.26 ± 0.58	1.31 ± 0.64	0.674
(n = 102 patients)	Diagnostic acceptability	1.24 ± 0.47	1.17 ± 0.42	0.214

Note—Values are given as mean ± standard deviation. SSSP = standard-speed, standard-pitch; HSHP = high-speed, high-pitch.

## Data Availability

The data presented in this study may be provided on reasonable request from the corresponding author.

## Supplementary Materials

The following supporting information can be downloaded at: https://www.mdpi.com/article/10.3390/medicina60081301/s1, Table S1: Grading scale for qualitative analysis of CT examination; Table S2: Mean values for the lung nodule attenuation, volume, max 2D diameter, and short axis diameter.
