# Supplementary material for: Increased Scan Speed and Pitch on Ultra-Low-Dose Chest CT: Effect on Nodule Volumetry and Image Quality
Source: Medicina (Kaunas). 2024 Aug 12;60(8):1301. doi: 10.3390/medicina60081301 (PMC11356370; doi:10.3390/medicina60081301)
Supplement: Supplementary file 1 [file medicina-60-01301-s001.zip › medicina-3112757-supplementary.pdf]

Supplementary Tables.

Table S1. Grading scale for qualitative analysis of CT examination

Scale	Nodule detection confidence	Nodule contour delineation	Streak artifacts	Overall diagnostic acceptability
1	Completely confident	Completely clear edge	None	Superior
2	Average confidence	Average edge	Mild artifacts-not relevant for diagnosis	Average
3	Poor confidence	Less clear edge-Poorer than average	Marked artifacts	Suboptimal
4	Not detected	Blurry margin	Severe artifacts	Unacceptable

Table S2. Mean values for the lung nodule attenuation, volume, max 2D diameter, and short axis diameter.

Nodule characteristics	SSSP	HSHP	P-value
Attenuation (HU)	-158.2 ± 226.1	-158.4 ± 224.5	0.978
3D Volume (mm3)	1378.7 ± 2042.2	1375.8 ± 2053.1	0.830
Max 2D diameter (mm)	12.6 ± 7.1	12.60 ± 7.2	0.887
Short axis diameter (mm)	8.6 ± 4.6	8.5 ± 4.5	0.453

Note. – Data are expressed as the mean ± standard deviation
